# Dr. Wm. H. Parkhurst

**Published:** 1901-02

**Authors:** 


Dr. Wm. H. Parkhurst, of Frankfort, N.Y., died January 22, 1901, aged 88 years. He was one of the oldest physicians in the Mohawk Valley, having been in constant practice since 1839, when he graduated at Geneva Medical College.

